# Engagement and Effectiveness of a Healthy-Coping Intervention via Chatbot for University Students During the COVID-19 Pandemic: Mixed Methods Proof-of-Concept Study

**DOI:** 10.2196/27965

**Published:** 2021-05-28

**Authors:** Silvia Gabrielli, Silvia Rizzi, Giulia Bassi, Sara Carbone, Rosa Maimone, Michele Marchesoni, Stefano Forti

**Affiliations:** 1 Digital Health Lab Fondazione Bruno Kessler Trento Italy

**Keywords:** mobile mental health, chatbots, anxiety, stress, university students, digital health, healthy-coping intervention, COVID-19

## Abstract

**Background:**

University students are increasingly reporting common mental health problems, such as stress, anxiety, and depression, and they frequently face barriers to seeking psychological support because of stigma, cost, and availability of mental health services. This issue is even more critical in the challenging time of the COVID-19 pandemic. Digital mental health interventions, such as those delivered via chatbots on mobile devices, offer the potential to achieve scalability of healthy-coping interventions by lowering cost and supporting prevention.

**Objective:**

The goal of this study was to conduct a proof-of-concept evaluation measuring the engagement and effectiveness of Atena, a psychoeducational chatbot supporting healthy coping with stress and anxiety, among a population of university students.

**Methods:**

In a proof-of-concept study, 71 university students were recruited during the COVID-19 pandemic; 68% (48/71) were female, they were all in their first year of university, and their mean age was 20.6 years (SD 2.4). Enrolled students were asked to use the Atena psychoeducational chatbot for 4 weeks (eight sessions; two per week), which provided healthy-coping strategies based on cognitive behavioral therapy, positive psychology, and mindfulness techniques. The intervention program consisted of conversations combined with audiovisual clips delivered via the Atena chatbot. Participants were asked to complete web-based versions of the 7-item Generalized Anxiety Disorder scale (GAD-7), the 10-item Perceived Stress Scale (PSS-10), and the Five-Facet Mindfulness Questionnaire (FFMQ) at baseline and postintervention to assess effectiveness. They were also asked to complete the User Engagement Scale–Short Form at week 2 to assess engagement with the chatbot and to provide qualitative comments on their overall experience with Atena postintervention.

**Results:**

Participants engaged with the Atena chatbot an average of 78 (SD 24.8) times over the study period. A total of 61 out of 71 (86%) participants completed the first 2 weeks of the intervention and provided data on engagement (10/71, 14% attrition). A total of 41 participants out of 71 (58%) completed the full intervention and the postintervention questionnaires (30/71, 42% attrition). Results from the completer analysis showed a significant decrease in anxiety symptoms for participants in more extreme GAD-7 score ranges (*t*_39_=0.94; *P*=.009) and a decrease in stress symptoms as measured by the PSS-10 (*t*_39_=2.00; *P*=.05) for all participants postintervention. Participants also improved significantly in the *describing* and *nonjudging* facets, based on their FFMQ subscale scores, and asked for some improvements in the user experience with the chatbot.

**Conclusions:**

This study shows the benefit of deploying a digital healthy-coping intervention via a chatbot to support university students experiencing higher levels of distress. While findings collected during the COVID-19 pandemic show promise, further research is required to confirm conclusions.

## Introduction

Increased numbers of adults, particularly university students, are experiencing symptoms of stress, anxiety, and depression [1,2], which are exacerbated by the recent restrictions introduced because of the COVID-19 pandemic [3,4]. In addition, up to 74% of adults experience their first onset of a mental health diagnosis before the age of 24 years [5]. However, about three-quarters of college students who are in need of clinical services do not access them [6]; this is because they have low mental health literacy and do not recognize a need for treatment [7], but also because of the high cost of treatment, low availability, or attitudinal barriers, such as perceived stigma [8,9]. In recent years, wider access to digital technology and mobile phones has presented new opportunities for overcoming these barriers, by offering the possibility of delivering digital mental health interventions in a more scalable and convenient way [10,11].

Empirical studies on evidence-based digital interventions for mental health, including internet-based cognitive behavioral therapy (CBT), have shown that these interventions are effective, feasible, and acceptable to users [12-14], although some limitations have been found, mainly regarding low engagement by users and low completion rates [15,16]. The integration of human coaching and support in digital mental health interventions can help improve adherence and behavior change outcomes [17,18], although this may reduce the scalability of such solutions. The design and deployment of conversational agents, such as chatbots, as virtual coaching solutions to deliver psychoeducational interventions for mental health and well-being have so far been shown to be ideal in maintaining the intuitiveness and naturalness of dialogue-based interaction, while exploiting the benefits of full automation [19,20]. These solutions seem to be particularly interesting to deploy at the time of the COVID-19 pandemic, when restrictions to face-to-face social encounters and interactions make it even more difficult to access human psychological support.

The use of chatbots for digital mental health interventions has attracted interest in the design community. A growing number of studies are reporting their acceptability and feasibility for users [20-22], as well as their effectiveness in reducing perceived stress [16-25] and abnormal eating behavior [26], thereby improving symptoms of anxiety [10,23-25], depression [10,24,25], and insomnia [27]. Like previous work and meta reviews have found on digital mental health interventions for the general adult population and for university students, a main limitation of these studies is their exclusive focus on randomized controlled trials, which prevents a full understanding of the challenges regarding user engagement, uptake, and adoption of these solutions [15-28]. More research is needed to understand the user experience (UX) and engagement with digital mental health interventions, to not only prove their clinical efficacy but also to facilitate their successful implementation in real-world settings [25-29].

The objective of this study was to assess the levels of engagement by university students and effectiveness of their interactions with a psychoeducational intervention delivered by the Atena chatbot over 1 month, in order to improve their coping and resilience skills during the COVID-19 pandemic. The study design was based on a mixed methods approach and encompassed two phases of the ORBIT (Obesity-Related Behavioral Intervention Trials) framework [30] for intervention design (Phase I) and preliminary testing (Phase IIa). In Phase I, the intervention, targets, and components were defined in order to specify their clinically relevant effect on users and to refine the intervention components. In Phase IIa, a proof-of-concept implementation of the digital intervention and chatbot was realized and preliminary testing was done for engagement and effectiveness with a convenience sample of university students. We hypothesized that use of the Atena chatbot over a 1-month period would lead to a reduction in symptoms of stress and anxiety and would prove to be engaging and acceptable to use by students.

To our knowledge, this is the first study to investigate the potential effect of a healthy-coping chatbot intervention during the COVID-19 pandemic, a time when the empowerment gained from stress management skills is much needed by the adult population, particularly university students.

## Methods

### The Atena Chatbot Design

Atena is a chatbot that delivers psychoeducational content in order to coach users in using coping strategies and improving their mental well-being by means of conversational dialogues with the coach Atena and audiovisual educational materials. It is accessible for free on the Telegram messaging app and is available on mobile or desktop devices. The chatbot was built using JavaScript and was developed by the Digital Health Lab at Fondazione Bruno Kessler (FBK) research center.

The digital mental health intervention delivered by Atena is aimed at improving users’ well-being by raising self-awareness about one’s thoughts and emotions; it does this by suggesting effective coping strategies that can be adopted when facing typical stressful situations, thus promoting mental well-being and preventing mental distress. The full program consists of eight short sessions, each lasting about 10 minutes, delivered twice a week for 4 weeks. Each session is initiated by the chatbot on a scheduled plan decided by the user during the first session. Users are invited by the chatbot to fill in web-based versions of psychological symptom questionnaires at baseline and postintervention, as well as a user engagement scale at the end of week 2.

**Figure 1:**
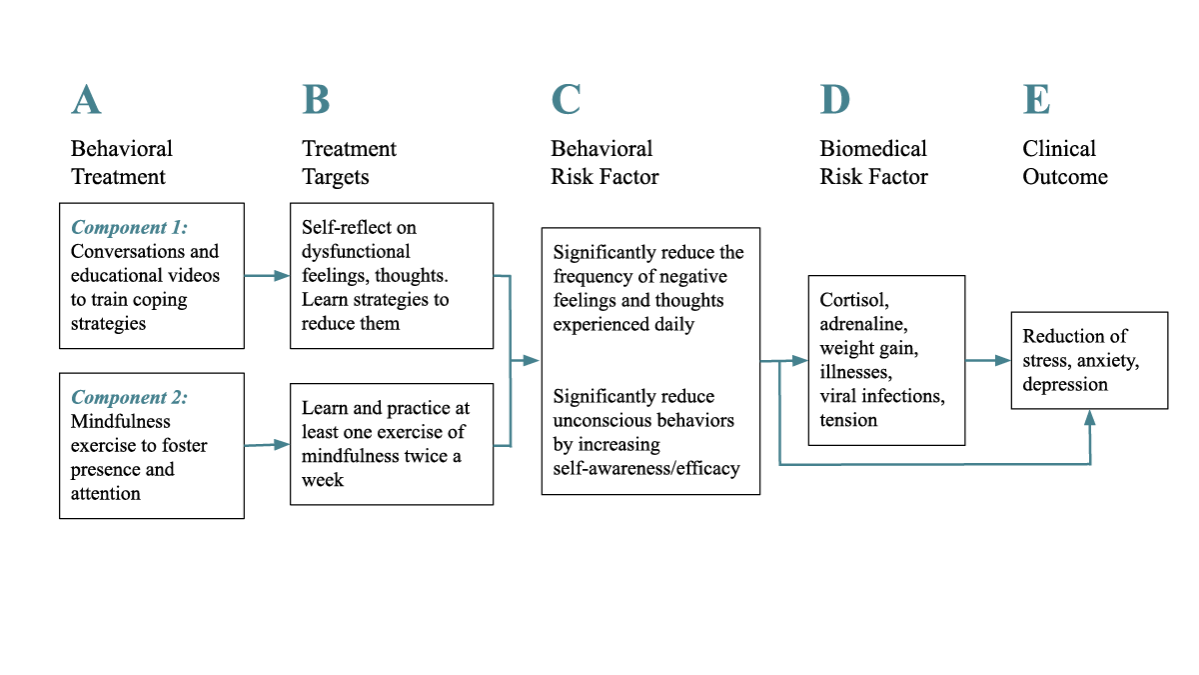
Definition of the healthy-coping behavioral intervention and clinical outcome.

The conversations between Atena and the user are informed by evidence-based approaches and intervention strategies from positive psychology and CBT, including psychoeducation on self-awareness and self-efficacy, conflict resolution, assertive communication, and practical exercises on mindfulness delivered at the end of each session [31,32]. These intervention strategies based on positive psychology, CBT, and mindfulness practices have been recently deployed by fully automated conversational agents targeting anxiety, stress, and depression, with promising results in terms of efficacy and acceptability by users [10,16,24,25]. The aim of the conversations is to trigger and support the user in self-reflecting on personal thoughts and emotions experienced in daily stressful settings, in learning how to best deploy more functional strategies to overcome difficulties, and in better managing stress and anxiety. The intervention program, including the behavioral and clinical targets, as well as the audiovisual content, were originally developed by a team of three clinical psychologists to fit the needs of the general adult population facing stress and anxiety challenges posed by the COVID-19 pandemic (Figure 1). A refinement of the conversations and video materials was then performed by the same psychologists in collaboration with two UX and behavior change experts in the design team, in order to adapt the language, videos, and chatbot interaction to the needs of the students in the target group.

**Figure 2:**
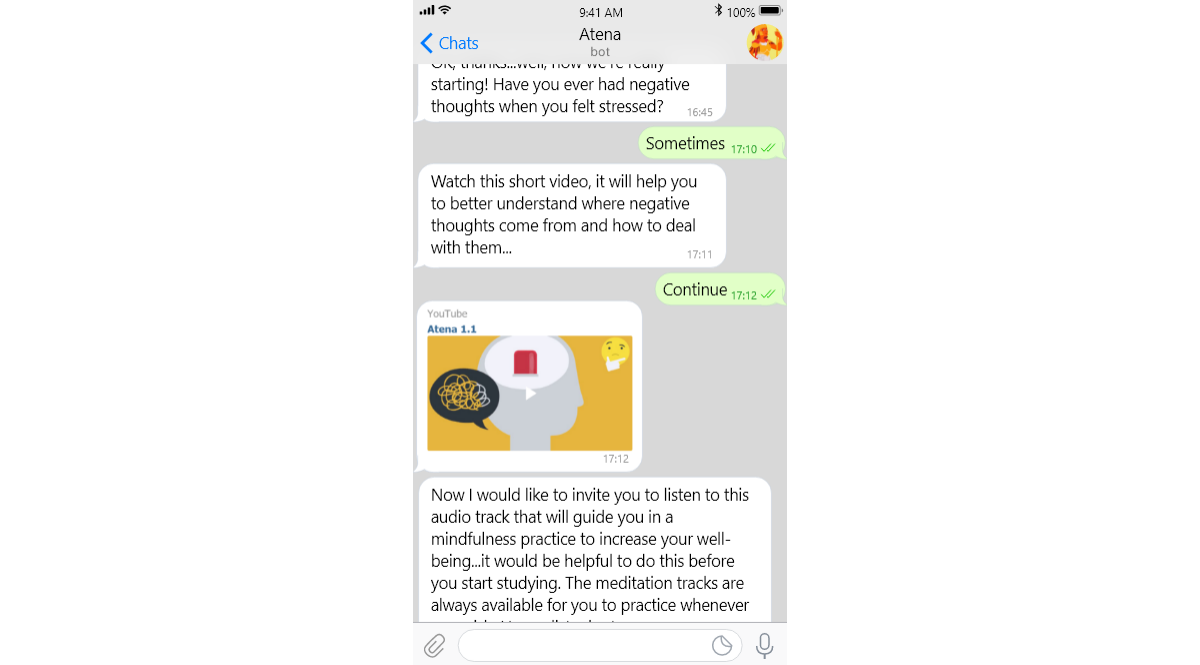
Screenshot from session 1 of the Atena chatbot intervention.

The Atena chatbot always starts the conversation on the scheduled date and time for that session, and the user replies by choosing among a predefined set of answer options. In this way, the conversation flow can be customized to sound more relevant and empathic to the user’s answers. Each session starts with a short psychoeducational video cartoon, representing typical challenging situations experienced by young characters and the corresponding strategies adopted to cope with them; they mimic relevant situations experienced by the target users, fostering their identification with those situations and their learning. In the final part of the session, the chatbot invites the user to perform a mindfulness exercise to focus her sense of presence and attention by following a coaching voice provided through an audio track (Figure 2).

Upon enrollment, users were made aware that the Atena chatbot was not intended to replace professional mental health treatment, but that it was a prototyped digital tool designed to support psychoeducational interventions and was going through preliminary testing in this study. In the first session, an introductory video cartoon was presented to the users by the Atena chatbot to explain the main features, applications, and limitations of chatbot technology, in order to facilitate the creation of appropriate expectations toward the digital tool and intervention tested.

### Participants

The Atena chatbot was voluntarily accessed and used by a recruited convenience sample of 71 university students; students ranged in age from 18 to 34 years (mean 20.6, SD 2.4) and the group consisted of 68% females (48/71). Students attended a human-computer interaction course in the first year of a bachelor’s degree program at the University of Trento, Italy; they were recruited and invited by SG and RM to access the chatbot via the messaging app Telegram. Participation was on a voluntary basis and the inclusion criteria were as follows: (1) being a university student in their first academic year and (2) owning a smartphone with a Telegram account. Atena was designed to offer coaching conversations, audiovisual materials, and mindfulness meditation in order to improve coping skills and well-being. Students used Atena between mid-October and November 2020, a period affected by the second wave of the COVID-19 pandemic in Italy, with restrictions to citizens’ mobility, social distancing, and blended learning recommended at the university. All users were Italian speakers located in the northeast of Italy.

### Measures

#### Perceived Stress Scale

The 10-item Perceived Stress Scale (PSS-10) is a brief self-report instrument containing 10 items rated on a 5-point Likert scale, ranging from 0 (never) to 4 (very often). The PSS-10 measures the perception of stress (ie, the degree to which situations are appraised as stressful) by asking respondents to rate the frequency of their thoughts and feelings related to situations that occurred recently [33] (eg, “In the last month, how often have you felt nervous and stressed?”). Levels of stress are determined based on scores as follows: low (0-13), moderate (14-26), and high (27-40). The PSS-10 is one of the most widely used psychological instruments, reporting good psychometric properties. In this study, the Cronbach α was .84.

#### Generalized Anxiety Disorder Scale

The 7-item Generalized Anxiety Disorder scale (GAD-7) [34] is a 7-item self-report scale based on a 4-point Likert scale, ranging from 0 (not at all) to 3 (nearly every day). The GAD-7 is used to assess anxiety symptoms over the past 2 weeks (eg, “How often have you been bothered by feeling afraid something awful might happen?”). The total scores are separated into the following four categories to determine the anxiety symptom levels: none (0-4), mild (5-9), moderate (10-14), and severe (≥15) [23,24]. In this study, the Cronbach α was .86.

#### Five-Facet Mindfulness Questionnaire

The Five-Facet Mindfulness Questionnaire (FFMQ) [35] is a 39-item self-report measure, evaluated on a 5-point Likert scale, ranging from 1 (never or very rarely true) to 4 (very often or always true); the questionnaire assesses the tendency to be mindful in daily life. The five facets are as follows: (1) observing (8 items; eg, “When I’m walking, I deliberately notice the sensations of my body moving”), (2) describing (8 items; eg, “I’m good at finding words to describe my feelings”), (3) acting with awareness (8 items; eg, “When I do things, my mind wanders off and I’m easily distracted”), (4) nonjudging (8 items; eg, “I criticize myself for having irrational or inappropriate emotions”), and (5) nonreactivity (7 items; eg, “I perceive my feelings and emotions without having to react to them”). Individual facet scores range from 8 to 40—except for the nonreactivity facet, which ranges from 7 to 35—with higher scores indicating more mindfulness. The sum of the direct- and reverse-scored items gives a total score that ranges from 39 to 195. In this study, the Cronbach α values were .74 for observing, .92 for describing, .85 for acting with awareness, .91 for nonjudging, .79 for nonreactivity, and .84 for the total score.

#### User Engagement Scale–Short Form

The User Engagement Scale–Short Form (UES-SF) [36] is a self-report measure comprised of 12 items rated on a 5-point Likert scale, ranging from 1 (strongly disagree) to 5 (strongly agree). The UES-SF measures the main determinants of adherence and, in particular, it assesses four factors: (1) perceived usability (eg, “Using Atena was frustrating”), (2) aesthetic appeal (eg, “Atena appealed to my senses”), (3) focused attention (eg, “I lost myself in this experience”), and (4) reward (eg, “This experience was rewarding”). The *reward* factor is a summary of three factors from the original User Engagement Scale (UES): *endurability*, a measure of how successful the interaction was and the likelihood of recommending the app to others; *novelty*, a measure of curiosity and interest; and *felt involvement*, a measure of the feeling of being “drawn in” and having fun [36]. A total score is calculated and scores for each of the four subscales are calculated by adding scores for all the items related to their factor and dividing them by the total items.

A back-translation was conducted; thus, all scales were translated into Italian and validated for use within this population. In this study, the Cronbach α values were .66 for perceived usability, .66 for focused attention, .52 for aesthetic appeal, .87 for reward, and .83 for the total scale.

### Procedures

After signing and submitting their digital consent form, users were instructed on how to fill in the web-based versions of the PSS-10, the GAD-7, and the FFMQ questionnaires, by using an alphanumeric pseudonymization code decided by them, and how to access the Atena chatbot on the Telegram app. In the first session with the chatbot, users were welcomed and provided with the introductory video on chatbot technology. They were also asked to set a desired day and time for their two weekly sessions with Atena, according to their preferences. The chatbot prompted the user to start a session at the scheduled day and time, but the user was free to pause, continue, or discontinue the session at any time.

At the end of week 2 (session 4), the users were invited by the chatbot to fill in the UES-SF questionnaire to assess their early engagement with the intervention and submit any free-text comments about their UX with the chatbot during the first 2 weeks of interaction.

At the end of week 4 (session 8), the users were invited by the chatbot to again fill in the PSS-10, GAD-7, and FFMQ questionnaires. The chatbot thanked them for their participation in the study and recommended that they continue engaging with the psychoeducational content that had been delivered, in order to improve their coping skills. A total of 4 weeks after the end of the study, participants were also asked to fill in a brief online survey to report on what they most liked and disliked about their experience with Atena, and whether they had continued to practice any of the exercises provided during the intervention with or without the support of Atena in the last 4 weeks. No monetary incentive was provided for participating in this study.

### Ethics and Informed Consent

The study was reviewed and approved by the FBK Institutional Ethics Board since it involved a nonclinical population. Participants indicated their consent for their pseudonymized data to be used for research purposes after reading an information sheet. All study data were collected by the Digital Health Lab of FBK. Because of deidentification of all data transmitted between the Atena chatbot and the user, usage data were not linked to specific research participants and were, therefore, reported in an aggregated format.

### Statistical Analyses

Statistical analyses were performed using R, version 4.0.0 (The R Foundation) [37], and SPSS Statistics, version 24.0 (IBM Corp) [38]. The Shapiro test was carried out to evaluate the normal distribution of the variables included in this study, such as the GAD-7 and the PSS-10 questionnaire scores (ie, anxiety and stress symptoms, respectively), the total score and the five subscale scores of the FFMQ, and the UES-SF questionnaire score.

The main descriptive analyses (ie, mean, standard deviation, and frequencies) were performed in order to assess the demographic characteristics of the overall sample (ie, age and gender), as well as the GAD-7 and the PSS-10 scores (ie, anxiety and stress symptoms, respectively), the total score and the five subscale scores of the FFMQ, and the UES-SF questionnaire score.

A paired-samples *t* test was conducted to evaluate differences between pre- and postintervention scores concerning the GAD-7 and the PSS-10 (ie, anxiety and stress symptoms, respectively) as well as the total score and the five subscale scores of the FFMQ. A *P* value equal to or less than .05 was considered statistically significant.

An independent-samples *t* test was conducted in order to evaluate differences between pre- and postintervention findings considering two clusters of users’ symptoms, extreme versus moderate ranges, as follows: (1) minimal and severe and (2) mild and moderate referring to the GAD-7 and the PSS-10 questionnaire scores. A *P* value equal to or less than .05 was considered statistically significant.

Participants’ responses to open-ended questions from the online final survey were analyzed by SR and SC using thematic analysis and were reported as frequencies. Data were analyzed thematically using an inductive, data-driven approach guided by the procedure outlined in Braun and Clarke [39]. Data codes were generated systematically, then collated into themes and applied to the entire data set to generate frequencies.

## Results

### Participants’ Questionnaire Scores by Gender

All the variables included in the analyses were normally distributed. As displayed in Table 1, at baseline, the overall sample (N=71) showed a mean that was close to the moderate range regarding the GAD-7 and the PSS-10 scores. More specifically, regarding the level of anxiety measured with the GAD-7, 35% (25/71) of students were in the *mild* range (ie, score of 5-9), 31% (22/71) were in the *moderate* range (ie, score of 10-14), and 20% (14/71) were in the *severe* range (ie, score of ≥15). Only 14% (10/71) of students were in the *minimal* range (ie, score of 0-4). Regarding anxiety symptoms for each gender, the analyses show that males experienced mild anxiety symptoms, while females experienced moderate anxiety symptoms.

Regarding stress symptoms evaluated with the PSS-10, 65% (46/71) of the sample were in the *moderate* range (ie, score of 14-26), 7% (5/71) were in the *low* range (ie, score of 0-13), and 28% (20/71) were in the *high* range (ie, score of 27-40). Both males and females displayed moderate stress symptoms.

Regarding the FFMQ scores, participants had an average total mindfulness score of 119.96 (SD 16.99); males scored higher (mean 126.26, SD 19.22) than females (mean 116.93, SD 15.10).

**Table 1:** Participants’ questionnaire scores by gender.

Questionnaire		Questionnaire score, mean (SD)		
		Overall sample (N=71)	Males (n=23)	Females (n=48)
GAD-7^a^		9.92 (4.88)	9.26 (4.66)	10.23 (5.01)
PSS-10^b^		22.46 (6.68)	20.78 (6.65)	23.27 (6.61)
**FFMQ^c^**				
	Observing facet	23.54 (5.81)	23.30 (5.78)	23.15 (5.89)
	Describing facet	23.55 (7.16)	25.00 (7.11)	22.85 (7.15)
	Act with awareness facet	25.85 (6.10)	26.30 (6.17)	25.63 (6.12)
	Nonjudging facet	26.04 (7.95)	28.13 (6.34)	25.04 (8.50)
	Nonreacting facet	18.44 (4.40)	20.39 (5.26)	17.50 (3.61)
	Total	119.96 (16.99)	126.26 (19.22)	116.93 (15.10)

^a^GAD-7: 7-item Generalized Anxiety Disorder scale. Scores for each item range from 0 (not at all) to 3 (nearly every day), and levels of anxiety are determined based on total scores as follows: none (0-4), mild (5-9), moderate (10-14), and severe (≥15).

^b^PSS-10: 10-item Perceived Stress Scale. Scores for each item range from 0 (never) to 4 (very often), and levels of stress are determined based on total scores as follows: low (0-13), moderate (14-26), and high (27-40).

^c^FFMQ: Five-Facet Mindfulness Questionnaire. Scores for each item within each facet range from 1 (never or very rarely true) to 4 (very often or always true). Individual facet scores range from 8 to 40—except for the nonreacting facet, which ranges from 7 to 35—with higher scores indicating more mindfulness, and total scores range from 39 to 195.

### Attrition

A total of 86% (61/71) of participants provided data on the user engagement questionnaire at the end of week 2, which represented an overall attrition rate of 14% (10/71). A total of 58% (41/71) of participants completed the postintervention questionnaire, which represented an overall attrition rate of 42% (30/71).

Dropout was higher among participants in the *minimal* and *mild* anxiety ranges of the GAD-7 questionnaire—50% (5/10) and 44% (11/25), respectively—and was lower in the *moderate* and *severe* anxiety ranges: 40% (9/22) and 35% (5/14), respectively. Moreover, dropout was higher among participants in the *moderate* and *low* stress ranges of the PSS-10 questionnaire—45% (21/46) and 40% (2/5), respectively—and lower in the *high* stress range: 35% (7/20).

### User Engagement With the Atena Chatbot

Table 2 shows the results of user engagement with the Atena chatbot as measured by the UES-SF questionnaire at week 2. A total of 61 out of 71 (86%) participants were in agreement with the perceived usability factor, which measured the affective (ie, frustration) and cognitive (ie, effortful) aspects as a result of the interaction. Participants answered in neutral ways regarding (1) the total UES-SF score; (2) the aesthetic appeal factor, which measured the sensory and visual appearance of the interface; and (3) the reward factor, which measured the hedonic aspects of experience, the felt involvement, the overall success of the interaction, and the willingness to engage with the chatbot in the future. Lastly, regarding the focused attention factor, which evaluated the focused concentration, absorption, and temporal dissociation, participants were in disagreement. Since participants were students attending a human-computer interaction course, their expectations regarding the UX and the quality of the user engagement with the chatbot might have been higher compared with students attending other higher education subjects. However, it should also be considered that participants were attending the first semester of their bachelor’s degree program, so their expertise in the field of technology design was still quite limited and comparable to that of other students in their age group.

**Table 2:** User engagement with the chatbot as measured by the UES-SF^a^ questionnaire.

UES-SF factor	Score (n=61), mean (SD)
Focused attention factor	2.73 (0.79)
Perceived usability factor	4.28 (0.66)
Aesthetic appeal factor	3.09 (0.65)
Reward factor	3.15 (0.84)
Total	3.15 (0.84)

^a^UES-SF: User Engagement Scale–Short Form. Scores for each item range from 1 (strongly disagree) to 5 (strongly agree). A total score is calculated and scores for each of the four subscales are calculated by adding scores for all the items related to their factor and dividing them by the total items.

### Preliminary Efficacy From Completer Analysis

There was a reduction of participants in the *severe* anxiety range of the GAD-7 at postintervention, from 20% (14/71) to 10% (4/41) (Table 3). Also, 7 participants who were above the clinical cutoff score for the GAD-7 of 8 or higher at baseline moved below this cutoff point at postintervention (7/41, 17%).

The independent-samples *t* test between pre- and postintervention between the two clusters of students—cluster 1 students had extreme symptoms and cluster 2 students had moderate symptoms—showed a significant difference (*t*_39_=0.94; *P*=.009) among anxiety ranges (ie, GAD-7 scores) in cluster 1, with a decrease of symptoms between preintervention (mean score 12.14, SD 6.88) and postintervention (mean score 10.07, SD 4.58). No other significant difference between pre- and postintervention GAD-7 scores was found.

In line with the GAD-7 results, the PSS-10 scores also showed an increase in the low stress range and a decrease in the high stress range (Table 4). This might indicate a positive effect of the intervention on participants who were in the more extreme stress level ranges compared to those in the intermediate stress ranges. Table 5 shows that the levels of stress symptoms (ie, PSS-10 scores) exhibited significant decreases (*t*_39_=2.00; *P*=.05) between pre- and postintervention. Moreover, the mean scores of the subscales *describing* and *nonjudging*, as well as the mean total score of the FFMQ, showed significant increases (*P*<.05) between pre- and postintervention.

**Table 3:** Classification of anxiety symptoms pre- and postintervention.

GAD-7^a^ anxiety level	Students preintervention (N=71), n (%)	Students postintervention (n=41), n (%)
Minimal	10 (14)	5 (12)
Mild	25 (35)	17 (41)
Moderate	22 (31)	15 (37)
Severe	14 (20)	4 (10)

^a^GAD-7: 7-item Generalized Anxiety Disorder scale.

**Table 4:** Classification of perceived stress symptoms pre- and postintervention.

PSS-10^a^ stress level	Students preintervention (N=71), n (%)	Students postintervention (n=41), n (%)
Low	5 (7)	5 (12)
Moderate	46 (65)	27 (66)
High	20 (28)	9 (22)

^a^PSS-10: 10-item Perceived Stress Scale.

**Table 5:** Paired-samples *t* test between pre- and postintervention (n=41).

Questionnaire		Questionnaire score, mean (SD)		Mean difference (SD)	*t* test (*df*=39)	*P* value^a^
		Preintervention	Postintervention			
GAD-7^b^		10.49 (4.62)	9.29 (0.72)	1.19 (4.14)	1.85	.07
PSS-10^c^		22.49 (6.52)	20.83 (0.97)	1.66 (5.30)	2.00	.05
**FFMQ^d^**						
	Observing facet	23.15 (5.84)	23.37 (6.50)	–0.22 (5.43)	–0.259	.80
	Describing facet	23.05 (7.29)	24.98 (6.03)	–1.92 (5.29)	–2.33	.03
	Act with awareness facet	26.15 (6.56)	26.12 (6.99)	0.02 (6.27)	0.03	.98
	Nonjudging facet	25.85 (7.78)	28.02 (7.46)	–2.17 (6.09)	–2.28	.03
	Nonreacting facet	18.41 (4.01)	18.66 (4.85)	–0.24 (4.55)	–0.34	.73
	Total	119.49 (16.56)	147.27 (19.67)	–27.78 (16.74)	–10.62	<.001

^a^*P* values were based on two-tailed *t* tests; values were significant at *P*<.05.

^b^GAD-7: 7-item Generalized Anxiety Disorder scale.

^c^PSS-10: 10-item Perceived Stress Scale.

^d^FFMQ: Five-Facet Mindfulness Questionnaire.

### Use of the Chatbot

**Figure 3:**
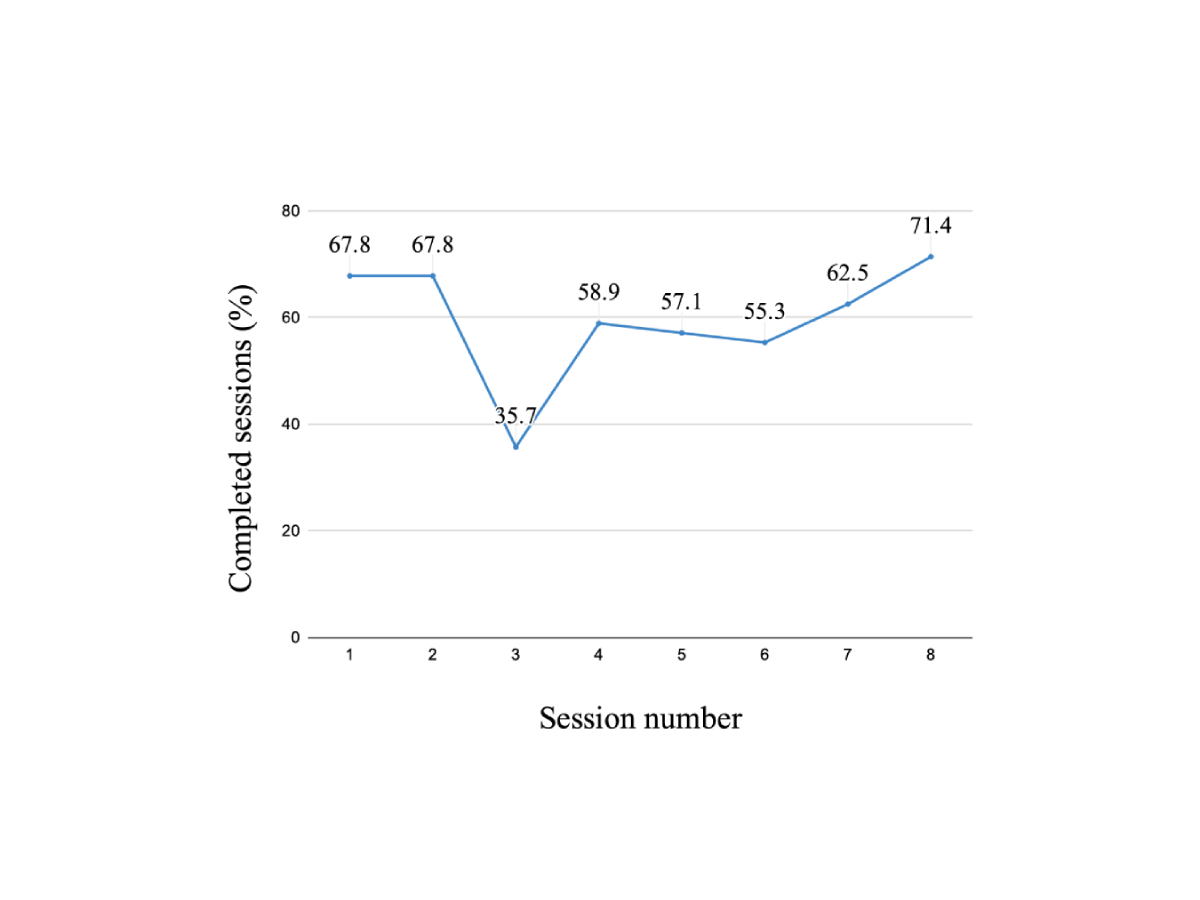
Overall percentage of completed sessions over the study's 4-week duration.

Participants interacted with the Atena chatbot an average of 78 times (SD 24.8; median 81; range 5-158) over the 4-week period. The average number of uncompleted sessions was 3.1 (SD 2.3) out of 8 overall sessions. Figure 3 shows a graph with the percentage of completed sessions over the 4-week period, showing that engagement and willingness to complete a session was higher during the first and the last weeks of the study.

### Qualitative Results

#### Overview

A total 17 out of 71 (24%) students—65% (11/17) females and 35% males (6/17)—provided qualitative data via the follow-up survey. Three main themes with related subthemes were identified (Table 6): (1) *content*, with the subthemes learning, reflection, multimedia, routine, mindfulness, motivation, originality, and repetitiveness; (2) *UX*, with the subthemes sense of reality, interaction, and flexibility; and (3) *tasks*, with the subthemes notification and availability.

#### Content

In terms of content, what students liked most was the availability of videos versus only having text-based dialogues in the interaction with the chatbot:

It was nice to have the possibility of accessing videos and not just using text, that might be boring.

However, one participant suggested that videos should be improved from a graphic design point of view:

What I liked less were the videos. They were interesting, but I think they should be improved in terms of graphics and visuals in order to be more engaging for users.

Some students appreciated the opportunity to learn new things—“I appreciated tips provided during the course, which were very interesting and also useful for learning new skills”—and to approach the mindfulness practice:

I find the bot an excellent first opportunity to start mindfulness practices, especially for those who - like me - can never find time to stop and breathe and have never tried anything like this before...

Some students also appreciated the originality of the exercises:

The originality of the exercises, in my opinion, is very important to foster change in people and help them.

One student suggested that the chatbot questions should be more varied in format so as not to be too repetitive:

In my opinion, questions should change over time: “how are you” should be asked in a more nuanced way, otherwise it sounds repetitive. I suggest, if possible, to vary the dialogues with the user, especially the welcoming messages.

#### User Experience

Regarding the UX, one student reported that what they liked most was the feeling of real-life interaction with the chatbot:

First of all, I appreciated the continuity of the short course with the chatbot, the interactions were well thought out and articulated, it felt like chatting with a real person.

Some students, however, reported some criticism regarding the user-chatbot interaction and gave some suggestions for future improvements:

The interaction with Atena should be more personalized based on the user’s answers, sometimes the answering options did not take into account the different nuances of mood.

#### Tasks

In the task theme, participants’ remarks mainly concerned the chatbot notifications and lack of reminders to resume a session when the user was interrupted by some other task:

I often didn’t have time to watch the videos at the scheduled time, then I forgot to resume them because there was no reminder.

The possibility of having the materials provided by Atena available in the chat was much appreciated:

I can watch videos again whenever I like.

A total of 8 out of the 17 (47%) students who provided qualitative data (ie, 4 females and 4 males) reported that they accessed the Atena videos and materials again after the 1-month study duration.

**Table 6:** Themes, subthemes, and quotes by participants.

Main theme and subthemes		Participant quotes
**Content**		
	Learning	“I enjoyed learning about new forms of interaction and their applications.” (Participant #3) “I appreciated tips given during the course, since they were very interesting and also useful for learning new skills.” (Participant #10)
	Reflection	“Reported topics were all interesting and quick to address. I spent very little time with the chatbot but I think I got some food for thought.” (Participant #1)
	Multimedia	“I liked the guided meditation videos.” (Participant #5) “I really liked the relaxation videos.” (Participant #7) “It was nice to have the possibility to access videos and not just texts, that can be boring.” (Participant #8) “I enjoyed the guided meditation videos.” (Participant #13) “What I liked most were the mindfulness and motivational videos.” (Participant #16) “Videos were long and I often tended to quit them before the end.” (Participant #3) “Videos often recommended walking or covering distances, which made it difficult to perform the task for people like me who live in a small indoor place (I often did not have the possibility or desire to move outdoors).” (Participant #9) “What I liked less was the videos. They were interesting, but I think they should be improved in terms of graphics and visuals in order to be engaging for users.” (Participant #10)
	Routine	“Unfortunately receiving videos for me had become a pleasant habit, so when it went over it looked like a sudden interruption to me.” (Participant #16)
	Mindfulness	“Breathing tips, calm tone of voice put me in a good mood, dialogues were motivating and relaxing.” (Participant #9) “I find the bot an excellent first opportunity to practice mindfulness, especially for those who - like me - can never find the time to stop and breathe and have never tried anything like this before.” (Participant #13)
	Motivation	“Low incentive.” (Participant #2) “It didn’t appeal to me so much, so I struggled to be constant.” (Participant #15)
	Originality	“The originality of the exercises, in my opinion, it is very important to foster a change in people to help them.” (Participant #6)
	Repetitiveness	“In my opinion, questions should vary over time: ‘how are you’ should be asked in a more nuanced way, otherwise it sounds repetitive. I suggest, if possible, to vary the dialogues with the user, especially the welcoming messages.” (Participant #16)
**User experience**		
	Sense of reality	“First of all, I appreciated the continuity of the short course with the chatbot, the interactions were well thought out and articulated, it felt like chatting with a real person.” (Participant #1)
	Interaction	“I would have liked to have more dialogues with the chatbot and less external interaction (YouTube video).” (Participant #1) “I would prefer to interact and chat whenever I wish, and not only on fixed days.” (Participant #8) “The interaction with Atena should be more personalized based on the user’s answers, sometimes the answering options did not take into account the different nuances of mood.” (Participant #9) “Interactions could only take place on the days agreed upon at the outset.” (Participant #13)
	Flexibility	“I really liked being able to have flexibility in the scheduling.” (Participant #2)
**Tasks**		
	Notification	“Notifications reminding people to take the test.” (Participant #4) “I feel satisfied. My only remark is about the notifications in the dialogue flow, which tended to be overlooked.” (Participant #4) “Not having time when the message arrived and then forgetting to do the exercises.” (Participant #5) “Atena sent me notifications when it was not suitable for me (despite my choosing of scheduling options) and then I forgot to do the activity.” (Participant #7) “It would be nice to specify at the very beginning when an activity requires places larger than a room or also to be outdoors.” (Participant #9) “I often didn’t have time to watch videos at the scheduled time, then I forgot to resume them because there was no reminder.” (Participant #15)
	Availability	“I can watch videos again whenever I like.” (Participant #11) “The scheduling options, in my opinion, should be more restrictive, or maybe deadlines could be set for some tasks.” (Participant #6) “The videos made it difficult for me to find enough time to watch them.” (Participant #12)

## Discussion

### Principal Findings

Results from this preliminary evaluation of the Atena chatbot intervention indicate that healthy-coping psychoeducation can be effectively deployed to university students and can have positive effects, especially on those who are more in need of psychological support to cope with stress and anxiety symptoms. Our results are in line with recent studies targeting the same population, showing that online stress management interventions are more effective for students with higher levels of stress, anxiety, and depression [40,41]. Higher engagement and lower attrition rates were also observed in those students in our sample who had more severe levels of anxiety at baseline. This is a very promising result for implementing future anxiety prevention and management solutions to be delivered during the COVID-19 pandemic and beyond. Results also showed a significant improvement in the capacity of participants to describe and accept their emotions, which can be an effect of the mindfulness practice and self-reflection elicited by the conversations with the chatbot. Training in these kinds of skills may be particularly needed by the university student population and could have positive effects on students’ mental well-being.

The baseline levels of stress and anxiety among our participants were significantly higher than those found in the same population of university students by previous research [42-44], as well as in the general population [45]. Previous studies analyzing the mental health of university students found lower levels of anxiety and stress symptoms, evaluated through the GAD-7 and the PSS-10 questionnaires, compared to those of our sample of university students [42-44]. This is not surprising, since the COVID-19 pandemic had a worsening effect on the general population and on university students in particular: a research study conducted on Italian university students to identify psychological consequences of the living conditions during the COVID-19 lockdown reported high levels of anxiety and stress, concentration disorders, psychosomatization, and, in several cases, reactivation of trauma and worsened sleep quality [46]. Moreover, from the time of our baseline assessment until the end of our study, the COVID-19 pandemic in Italy reached higher levels of infection, which required more severe restrictions to be introduced in schools and universities (ie, full online teaching) and in citizens’ everyday lives. It is likely that students experiencing more severe symptoms of anxiety felt more motivated to engage with the Atena chatbot and found it a more convenient solution to access psychoeducational support, by avoiding, at the same time, stigma and possible difficulties in accessing mental health services.

Overall, our quantitative and qualitative findings are aligned with recent chatbot evaluation studies [10,23-25] and will inform our design decisions for future developments. Results regarding attrition rates during the study, user engagement, as well as our qualitative findings suggest that our proof-of-concept intervention needs to be further refined to fully meet the requirements and preferences of the target users before being ready for randomized controlled trial evaluations. Our analysis indicates that the engagement and attractiveness of a chatbot-based mental health intervention for university students might wear off or reduce significantly after 2 weeks of interaction, requiring deeper levels of engagement through conversation and rewarding feedback from the chatbot in order to maintain users’ interest and commitment during the intervention. This might be particularly useful for supporting adherence of less motivated users, such as the ones with mild to moderate levels of stress and anxiety. Our study also helps shed light on what might be the ideal length, frequency, or intensity of digital mental health interventions for nonclinical populations. The duration and intensity of our intervention was sufficient to provide psychoeducational support to students without interfering too much with their daily life commitments, and it was also effective in triggering more self-reflection and mindfulness practice in the follow-up period. This might be interpreted as a signal of user empowerment and desired behavior change, although more research is needed to confirm this interpretation.

### Limitations

This study presents some limitations that affect the generalizability of the findings. It reports data from the preliminary evaluation of a proof-of-concept chatbot intervention targeting a homogeneous population of university students without a control group. Although the findings on user engagement and preliminary effectiveness of the intervention are promising and aligned with previous research, further testing by means of controlled trials should be conducted to confirm any conclusion about efficacy and to verify its maintenance at follow-up. However, the evidence presented from students’ responses and feedback to the intervention confirm the importance of deploying user-centered methodology in the iterative design and refinement of these interventions before investing additional resources in conducting more rigorous efficacy testing.

Another limitation is related to our method of collecting objective data on users’ engagement and interaction with the chatbot intervention during the study. Since our log data were deidentified, it was more difficult to assess any difference among users in how deeply they focused attention and self-reflected upon the psychoeducational videos’ contents and chatbot suggestions during each session. Although we could derive some information on users’ satisfaction with these contents from participants’ qualitative comments, a more complete and objective monitoring of users’ behavioral interactions with the intervention’s components would be preferable to deploy in future studies.

Finally, the study was conducted during the second wave of the COVID-19 pandemic in Italy, characterized by the introduction of increasingly more rigid restrictions to social behavior and educational practices that might have strongly impacted the mental well-being of our participants and reduced the positive effect of our intervention. However, the challenging contextual setting in which our intervention was deployed can also be considered a point of strength of the contribution provided by this study, offering interesting insights for the future wider deployment of digital mental health interventions in challenging conditions.

### Conclusions

This study further extends previous research on the use of chatbot-based interventions for healthy coping with stress, confirming their effectiveness in supporting university students experiencing higher levels of distress. Although the generalizability of the reported findings should be viewed with caution, since no control group was involved and the intervention was deployed during the COVID-19 pandemic, these preliminary findings are interesting for inspiring the future design of digital mental health interventions for university students and public health.

## Abbreviations

CBT: cognitive behavioral therapy
FBK: Fondazione Bruno Kessler
FFMQ: Five-Facet Mindfulness Questionnaire
GAD-7: 7-item Generalized Anxiety Disorder scale
ORBIT: Obesity-Related Behavioral Intervention Trials
PSS-10: 10-item Perceived Stress Scale
UES: User Engagement Scale
UES-SF: User Engagement Scale–Short Form
UX: user experience

## References

[1] Pinder-Amaker S, Bell C (2012). A bioecological systems approach for navigating the college mental health crisis. Harv Rev Psychiatry.

[2] Lipson SK, Lattie EG, Eisenberg D (2019). Increased rates of mental health service utilization by US college students: 10-year population-level trends (2007-2017). Psychiatr Serv.

[3] Le HT, Lai AJX, Sun J, Hoang MT, Vu LG, Pham HQ, Nguyen TH, Tran BX, Latkin CA, Le XTT, Nguyen TT, Pham QT, Ta NTK, Nguyen QT, Ho RCM, Ho CSH (2020). Anxiety and depression among people under the nationwide partial lockdown in Vietnam. Front Public Health.

[4] Tran BX, Nguyen HT, Le HT, Latkin CA, Pham HQ, Vu LG, Le XTT, Nguyen TT, Pham QT, Ta NTK, Nguyen QT, Ho CSH, Ho RCM (2020). Impact of COVID-19 on economic well-being and quality of life of the Vietnamese during the national social distancing. Front Psychol.

[5] Kessler RC, Amminger GP, Aguilar-Gaxiola S, Alonso J, Lee S, Ustün TB (2007). Age of onset of mental disorders: A review of recent literature. Curr Opin Psychiatry.

[6] Hunt J, Eisenberg D (2010). Mental health problems and help-seeking behavior among college students. J Adolesc Health.

[7] Eisenberg D, Golberstein E, Gollust SE (2007). Help-seeking and access to mental health care in a university student population. Med Care.

[8] Andrade LH, Alonso J, Mneimneh Z, Wells JE, Al-Hamzawi A, Borges G, Bromet E, Bruffaerts R, de Girolamo G, de Graaf R, Florescu S, Gureje O, Hinkov HR, Hu C, Huang Y, Hwang I, Jin R, Karam EG, Kovess-Masfety V, Levinson D, Matschinger H, O'Neill S, Posada-Villa J, Sagar R, Sampson NA, Sasu C, Stein DJ, Takeshima T, Viana MC, Xavier M, Kessler RC (2013). Barriers to mental health treatment: Results from the WHO World Mental Health surveys. Psychol Med.

[9] Fukuda CC, Penso MA, Amparo DMD, Almeida BCD, Morais CDA (2016). Mental health of young Brazilians: Barriers to professional help-seeking. Estud Psicol (Campinas).

[10] Fitzpatrick KK, Darcy A, Vierhile M (2017). Delivering cognitive behavior therapy to young adults with symptoms of depression and anxiety using a fully automated conversational agent (Woebot): A randomized controlled trial. JMIR Ment Health.

[11] Menezes P, Quayle J, Garcia Claro H, da Silva S, Brandt LR, Diez-Canseco F, Miranda JJ, Price LN, Mohr DC, Araya R (2019). Use of a mobile phone app to treat depression comorbid with hypertension or diabetes: A pilot study in Brazil and Peru. JMIR Ment Health.

[12] Torous J, Chan SR, Yee-Marie Tan S, Behrens J, Mathew I, Conrad EJ, Hinton L, Yellowlees P, Keshavan M (2014). Patient smartphone ownership and interest in mobile apps to monitor symptoms of mental health conditions: A survey in four geographically distinct psychiatric clinics. JMIR Ment Health.

[13] Davies EB, Morriss R, Glazebrook C (2014). Computer-delivered and web-based interventions to improve depression, anxiety, and psychological well-being of university students: A systematic review and meta-analysis. J Med Internet Res.

[14] Zhang MW, Ho RC (2017). Moodle: The cost effective solution for internet cognitive behavioral therapy (I-CBT) interventions. Technol Health Care.

[15] Torous J, Nicholas J, Larsen ME, Firth J, Christensen H (2018). Clinical review of user engagement with mental health smartphone apps: Evidence, theory and improvements. Evid Based Ment Health.

[16] Ly KH, Ly A, Andersson G (2017). A fully automated conversational agent for promoting mental well-being: A pilot RCT using mixed methods. Internet Interv.

[17] Johansson R, Andersson G (2012). Internet-based psychological treatments for depression. Expert Rev Neurother.

[18] Baumeister H, Reichler L, Munzinger M, Lin J (2014). The impact of guidance on internet-based mental health interventions — A systematic review. Internet Interv.

[19] Suganuma S, Sakamoto D, Shimoyama H (2018). An embodied conversational agent for unguided internet-based cognitive behavior therapy in preventative mental health: Feasibility and acceptability pilot trial. JMIR Ment Health.

[20] Vaidyam AN, Wisniewski H, Halamka JD, Kashavan MS, Torous JB (2019). Chatbots and conversational agents in mental health: A review of the psychiatric landscape. Can J Psychiatry.

[21] Gaffney H, Mansell W, Tai S (2019). Conversational agents in the treatment of mental health problems: Mixed-method systematic review. JMIR Ment Health.

[22] Gabrielli S, Rizzi S, Carbone S, Donisi V (2020). A chatbot-based coaching intervention for adolescents to promote life skills: Pilot study. JMIR Hum Factors.

[23] Fulmer R, Joerin A, Gentile B, Lakerink L, Rauws M (2018). Using psychological artificial intelligence (Tess) to relieve symptoms of depression and anxiety: Randomized controlled trial. JMIR Ment Health.

[24] Inkster B, Sarda S, Subramanian V (2018). An empathy-driven, conversational artificial intelligence agent (Wysa) for digital mental well-being: Real-world data evaluation mixed-methods study. JMIR Mhealth Uhealth.

[25] Daley K, Hungerbuehler I, Cavanagh K, Claro HG, Swinton PA, Kapps M (2020). Preliminary evaluation of the engagement and effectiveness of a mental health chatbot. Front Digit Health.

[26] Low TL, Ho R, Ho C, Tam W (2021). The efficacy of virtual reality in the treatment of binge-purging eating disorders: A meta-analysis. Eur Eat Disord Rev.

[27] Soh HL, Ho RC, Ho CS, Tam WW (2020). Efficacy of digital cognitive behavioural therapy for insomnia: A meta-analysis of randomised controlled trials. Sleep Med.

[28] Lattie EG, Adkins EC, Winquist N, Stiles-Shields C, Wafford QE, Graham AK (2019). Digital mental health interventions for depression, anxiety, and enhancement of psychological well-being among college students: Systematic review. J Med Internet Res.

[29] Fleming T, Bavin L, Lucassen M, Stasiak K, Hopkins S, Merry S (2018). Beyond the trial: Systematic review of real-world uptake and engagement with digital self-help interventions for depression, low mood, or anxiety. J Med Internet Res.

[30] Czajkowski SM, Powell LH, Adler N, Naar-King S, Reynolds KD, Hunter CM, Laraia B, Olster DH, Perna FM, Peterson JC, Epel E, Boyington JE, Charlson ME (2015). From ideas to efficacy: The ORBIT model for developing behavioral treatments for chronic diseases. Health Psychol.

[31] Seligman MEP (1998). Building human strength: Psychology's forgotten mission. APA Monitor.

[32] Beck JS (2011). Cognitive Behavior Therapy: Basics and Beyond. 2nd edition.

[33] Cohen S (1994). Perceived Stress Scale. Mind Garden.

[34] Spitzer RL, Kroenke K, Williams JBW, Löwe B (2006). A brief measure for assessing generalized anxiety disorder: The GAD-7. Arch Intern Med.

[35] Baer RA, Smith GT, Hopkins J, Krietemeyer J, Toney L (2006). Using self-report assessment methods to explore facets of mindfulness. Assessment.

[36] O’Brien HL, Cairns P, Hall M (2018). A practical approach to measuring user engagement with the refined user engagement scale (UES) and new UES short form. Int J Hum Comput Stud.

[37] R Core Team (2020). R 4.0.0 for Windows. The Comprehensive R Archive Network.

[38] (2016). IBM SPSS Statistics for Windows, Version 24.0. IBM Corp.

[39] Braun V, Clarke V (2006). Using thematic analysis in psychology. Qual Res Psychol.

[40] Coudray C, Palmer R, Frazier P (2019). Moderators of the efficacy of a web-based stress management intervention for college students. J Couns Psychol.

[41] Bower P, Kontopantelis E, Sutton A, Kendrick T, Richards DA, Gilbody S, Knowles S, Cuijpers P, Andersson G, Christensen H, Meyer B, Huibers M, Smit F, van Straten A, Warmerdam L, Barkham M, Bilich L, Lovell K, Liu ET (2013). Influence of initial severity of depression on effectiveness of low intensity interventions: Meta-analysis of individual patient data. BMJ.

[42] Mirón J, Goldberg X, López-Solà C, Nadal R, Armario A, Andero R, Giraldo J, Ortiz J, Cardoner N, Palao D (2019). Perceived stress, anxiety and depression among undergraduate students: An online survey study. J Depress Anxiety.

[43] Alghadir A, Manzar MD, Anwer S, Albougami A, Salahuddin M (2020). Psychometric properties of the Generalized Anxiety Disorder Scale among Saudi university male students. Neuropsychiatr Dis Treat.

[44] Anwer S, Manzar MD, Alghadir AH, Salahuddin M, Abdul Hameed U (2020). Psychometric analysis of the Perceived Stress Scale among healthy university students. Neuropsychiatr Dis Treat.

[45] Wang C, Tee M, Roy AE, Fardin MA, Srichokchatchawan W, Habib HA, Tran BX, Hussain S, Hoang MT, Le XT, Ma W, Pham HQ, Shirazi M, Taneepanichskul N, Tan Y, Tee C, Xu L, Xu Z, Vu GT, Zhou D, Koh BJ, McIntyre RS, Ho C, Ho RC, Kuruchittham V (2021). The impact of COVID-19 pandemic on physical and mental health of Asians: A study of seven middle-income countries in Asia. PLoS One.

[46] Savarese G, Curcio L, D'Elia D, Fasano O, Pecoraro N (2020). Online university counselling services and psychological problems among Italian students in lockdown due to Covid-19. Healthcare (Basel).

